# Retinal Vessel Flicker Light Responsiveness and Its Relation to Analysis Protocols and Static and Metabolic Data in Healthy Subjects

**DOI:** 10.3390/biomedicines13051201

**Published:** 2025-05-15

**Authors:** Dmitri Artemiev, Christophe Valmaggia, Scott Tschuppert, Konstantin Kotliar, Cengiz Türksever, Margarita G. Todorova

**Affiliations:** 1 Department of Ophthalmology, Cantonal Hospital St. Gallen, 9007 St. Gallen, Switzerlandmargarita.todorova@kssg.ch (M.G.T.); 2 Medical Faculty, University of Zurich, CH-8008 Zurich, Switzerland; 3 Department of Medical Engineering and Technomathematics, FH Aachen University of Applied Sciences, 52428 Juelich, Germany; 4 Department of Ophthalmology, VISTA Clinic, 4102 Binningen, Switzerland

**Keywords:** retinal vessel analyzer, retinal oxygen saturation, flicker light, metabolic structure–relationship

## Abstract

**Background:** The aim of this study was to assess the agreement between different analysis protocols for the determination of retinal vessel dilation response to flicker light (FL) and its relation to static and metabolic parameters of retinal vessels in healthy subjects. **Methods**: In total, 24 right eyes of 24 healthy controls (mean age: 36.04 ± SD 14.4 years) who underwent dynamic and static retinal diameter and oxygen saturation measurements on a Retinal Vessel Analyzer (RVA, Imedos, Jena, Germany) were included. Using repeated video analyses, responses to FL were measured with RVA. These measurements were conducted at three specific retinal locations: within the superotemporal area—within a distance of less than one optic disk (OD) diameter to optic nerve head (ONH) (group 1); greater than one OD diameter to ONH (group 2); and areas near the ONH within the VesselMap region (group 3). For comparability, the static and oxygen saturation parameters were also calculated in the superotemporal peripapillary area using the VesselMap tool of the RVA and were evaluated in relation to the corresponding dynamic area (group 3). **Results**: In all groups, the vascular FL response of arteries was less pronounced compared to venules (*p* = 0.0014). Even though FL responses (mean ± SD: FL-A; FL-V) in group 1 were more pronounced (3.36 ± 2.31; 4.42 ± 1.69) compared to those in group 2 (2.97 ± 2.40; 4.08 ± 1.55) and group 3 (2.84 ± 2.29; 4.21 ± 2.03), they did not reach statistically significant values. The mean flicker response of venules (VDil) in all groups showed negative correlations to the corresponding static parameter: central retinal venous equivalent (CRV) (*r* = −0.0437; *p* = 0.015). The mean flicker response of arteries (ADil) in all groups showed negative correlations to the corresponding metabolic parameter: arterio-venous oxygen extraction fraction (*r* = −0.101; *p* = 0.041). **Conclusions**: Our study confirms that the flicker light response, despite slight variations in its duration and location, allows for reliable measurements, proving the Retinal Vessel Analyzer to be a valuable diagnostic tool. Furthermore, we were able to highlight the relationship between the dynamic and metabolic components of retinal supply, which enables early diagnosis concerning the development of diseases within this spectrum.

## 1. Introduction

The condition of arterioles, capillaries, and venules in the microcirculation can provide insights into several underlying diseases, such as diabetes mellitus, arterial hypertension, and atherosclerosis [1,2,3]. In these diseases, disruptions in microcirculation reduce the oxygen supply to tissues, which can lead to serious complications and organ damage over time [4,5]. The function of the vessel endothelium is crucial in this process, and studies have shown that many systemic diseases negatively affect this function [6,7,8]. An eye examination is a unique and non-invasive way to examine vessels directly. Because of the similarity between retinal blood supply and cerebral circulation (due to their close physiological and embryological connection), observing retinal blood vessels can help detect early changes linked to systemic diseases [9,10,11].

There are already methods available for investigating microcirculation and endothelial function, such as flow-mediated dilation [12] and venous occlusion plethysmography [13]. However, these methods provide only limited information about the properties of the blood vessels. Current vascular analysis research is divided into static and dynamic methods. In static assessments, vessel diameter measurements are taken using fundus photography; afterwards, a software is used to calculate the central retinal arteriolar equivalent (CRA) and central retinal venous equivalent (CRV) [14,15,16]. The ratio of the CRA to CRV, known as the arteriovenous ratio (AVR), allows researchers to make predictions regarding the incidence of cardiovascular and cerebral events [17,18]. Static analysis is limited by measurement variability. Vessel size fluctuates with each pulse, and additional variability arises due to age-related differences [19]. Moreover, a single still image of the vessels does not provide information about the dynamics and functionality of microcirculation. This restricts our ability to observe real-time changes in blood flow and endothelial performance [20].

Dynamic vascular analysis offers a promising approach for investigating vascular behavior and endothelial function [21,22]. This method employs the principle of neurovascular coupling, where vascular responses are triggered by increased retinal metabolic demand after stimulation with flickering light [23,24,25]. The Retinal Vessel Analyzer (RVA) has become an important tool in this field, enabling the non-invasive measurement of retinal vessel dilation in response to light stimulation over time [26,27]. Numerous protocols and methodological variations for this procedure have been described in the literature by various research groups [21,28]. One of the most commonly applied measurement protocols involves an initial recording of baseline vascular response under constant retinal illumination. This is followed by three stimulation phases using flickering light at 12.5 Hz, each lasting at least 20 s, with a minimum interval of 80 s between stimulations [21].

The RVA’s high temporal resolution and sensitivity allow it to detect subtle changes in vessel diameter within seconds, making it particularly suitable for capturing rapid fluctuations in ocular perfusion parameters [29]. However, despite its advantages, the RVA has several limitations that affect its clinical and research applications. One major challenge lies in interpreting measurement results, which can be influenced by physiological factors such as baseline diameter fluctuations caused by vascular tone and arterial pulsation [30]. Additionally, variations in measurement protocols can impact the results, as the standard practice of averaging responses over multiple stimulation cycles may obscure the unique characteristics of individual cycles, potentially reducing the diagnostic sensitivity of the RVA [31]. Furthermore, there are limited data on the reproducibility of measurements across different variables. Some studies have evaluated the short-term and long-term coefficients of variation, revealing that the reproducibility of measurements is slightly better for veins compared to arteries [32,33,34]. To establish a comprehensive response profile for dynamic responses, several parameters need to be considered, including the point of maximum dilation, maximum constriction, dilation amplitude, and the time course of each reaction. These parameters can vary significantly among individuals, emphasizing the need for standardized protocols and further research to enhance the diagnostic and predictive value of the RVA in assessing vascular health [29,31,32].

Retinal examinations using flickering light stimulation enable non-invasive measurement of retinal vessel oximetry through dual-wavelength photographic technology [35]. The flicker light stimulation increases the metabolic demand of the retina, primarily due to the heightened activity of photoreceptors and glial cells. This metabolic response leads to elevated oxygen consumption, which subsequently increases retinal blood flow [36,37,38]. Retinal oximetry provides a simple yet effective method to evaluate metabolic state of the retina.

To accurately interpret the results from the Retinal Vessel Analyzer (RVA), especially in repeated measurements and long-term studies, it is important to evaluate how consistent and reliable the values obtained with this system are. Reproducibility is crucial to ensure the results can be trusted for clinical and research purposes. The goal of our study is to determine how reproducible dynamic measurements with flicker light stimulation are when using different protocols in healthy individuals. We also aim to compare static and metabolic parameters with dynamic ones to better understand their connections and clinical significance. By doing this, we hope to provide useful insights into the reliability of RVA measurements and their use in long-term monitoring and diagnostics. This is particularly important as RVAs are increasingly used to assess retinal microvascular health and its role in detecting both eye and systemic diseases.

## 2. Materials and Methods

The study was conducted in accordance with the principles outlined in the Declaration of Helsinki and received approval from the local institutional review board. All subjects were recruited between 2018 and 2022 from the ophthalmology department of our institution after an anamnesis-based evaluation of their health status and a comprehensive ophthalmological examination. Patients with eye diseases, optical impairments, or systemic conditions such as diabetes mellitus or arterial hypertension were excluded. Written informed consent was obtained from all participants prior to their inclusion in the study. Measurements were performed on 24 healthy subjects, with 24 right eyes analyzed (9 males, 15 females). Vessel diameter measurements were recorded both before and during light flicker stimulation. Pupil dilation of the selected eye was achieved using Tropicamide eye drops (Mydriaticum Dispersa, CIBA Vision, Switzerland) to ensure optimal visualization of the retinal vessels.

### 2.1. Retinal Vessel Imaging

Retinal vessel measurements were performed using the Retinal Vessel Analyzer (RVA; IMEDOS Systems UG, Jena, Germany), which integrates a modified fundus camera (Zeiss FF450, Carl Zeiss Meditec, Jena, Germany), a charge-coupled device camera, and software for data acquisition and analysis. The fundus camera, offering a 30° or 50° field of view, was used to acquire images for static vessel analysis, which were processed using the integrated VesselMap module of the RVA, and to record retinal images for dynamic assessment, with the integrated software enabling real-time analysis of vessel diameter changes. The RVA system captured vessel diameters at a time resolution of 25 readings per second, ensuring high temporal precision for dynamic assessments [39]. After a minimum of 20 min, four test–retest fundus images were obtained and optic disk-centered fundus images of the right eye were taken. The flicker light frequency was set at 12.5 Hz, alternating between illuminated and dark images, optimized for retinal stimulation. The superior temporal quadrant, identified in the literature as the most suitable and most frequently used site for dynamic measurements [40], was selected for analysis. In this quadrant, three predefined locations were examined. The respective vessels, all with diameters exceeding 100 μm, were manually selected on the live monitor. The measurement started after an initial time of 50 s with still illumination with three cycles of 20 s flickering light each followed an interruption of 80 s of recovery periods with still illumination. The system used observation light in the 530–600 nm wavelength range for optimal contrast between retinal vessels and surrounding tissue. Real-time data on vessel diameters were displayed graphically and numerically during the measurement, allowing continuous monitoring and immediate feedback [21].

**Figure 1:**
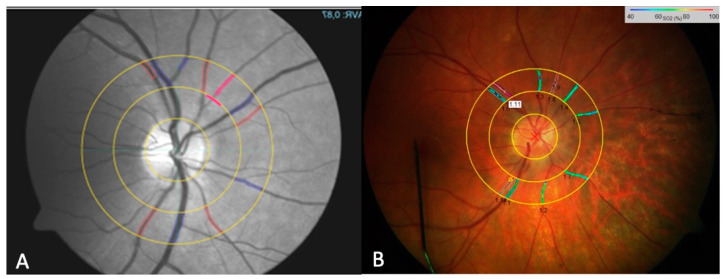
The fundus photo using the RVA for determining the parameters of static retinal vessel analysis with the VesselMap software (IMEDOS Systems, Jena, Germany). The marking of selected vessels—arteries (red) and veins (blue)—is performed semi-automatically. Once all visible vessels within the ring mask are marked, the parameters CRAE, CRVE, and AVR are automatically calculated (**A**). A representation of the retinal oximetry image, showing color-coded oxygen saturation (SO_2_) values of retinal vessels within the peripapillary annulus of a subject (**B**).

**Figure 2:**
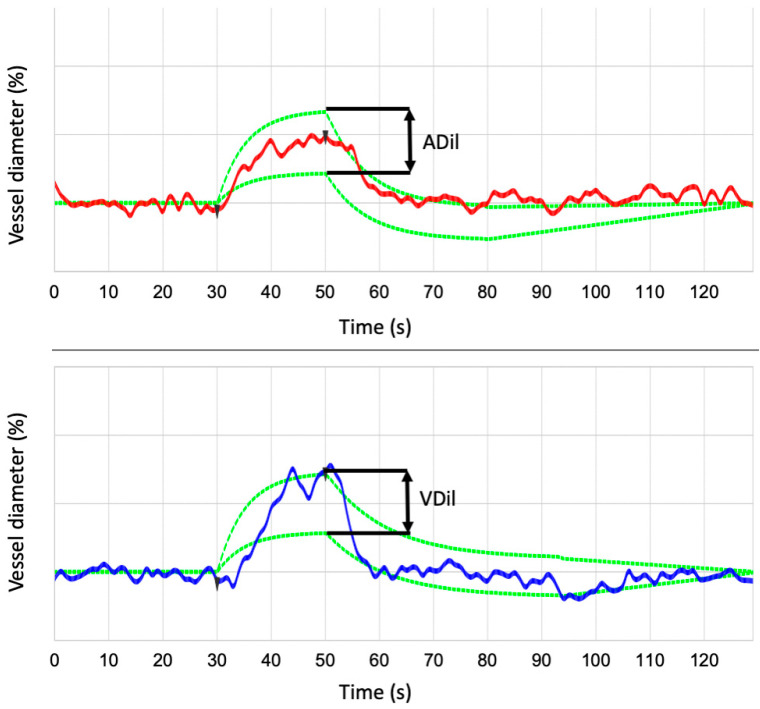
RVA internal data analysis for the arterial and venous segment. From three flicker cycles, an average cycle and a parameter of the flicker response with maximum vessel dilation for arteries (ADil) and veins (VDil) are calculated.

**Figure 3:**
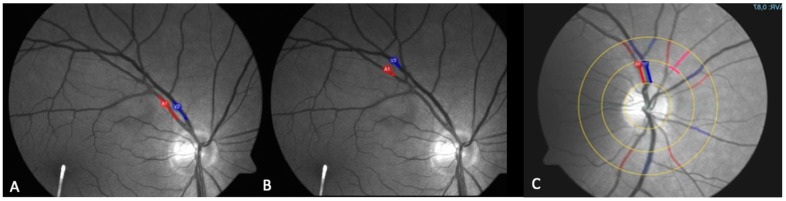
The fundus photograph taken using the RVA camera, with markings for the vein (blue) and artery (red) for group I (**A**), group II (**B**), and group III (**C**).

We gathered standard static analysis data using the VesselMap tool of the RVA (Figure 1A). The VesselMap software (RVA; IMEDOS Systems UG, Jena, Germany) semi-automatically selected the vessels and then calculated the central retinal artery (CRA) and central retinal vein (CRV) diameters, from which the arteriovenous ratio was calculated. In this way, the two static parameters describing the retinal vessel diameter of arteries (D-A) and veins (D-V) were also determined. Arterial dilation (ADil) and venous dilation (VDil), as dynamic parameters, were derived using the RVA software (RVA; IMEDOS Systems UG, Jena, Germany) during flicker light stimulation (Figure 2). These measurements were performed at each of the three predefined retinal locations for every individual subject. The measurement sites were determined based on their distance from the optic nerve head (ONH): vessels in group I were located within less than one optic disk (OD) diameter from the ONH (Figure 3A); group II included vessels at a distance greater than one OD diameter to ONH (Figure 3B); and group III comprised the area surrounding the ONH that corresponded with the VesselMap analysis region (Figure 3C). For Group III, measurements were specifically conducted within the VesselMap region to facilitate better correlation with the static vessel parameters, which were likewise obtained in this anatomical location.

Retinal vessel oximetry was performed using a spectrophotometric oximetry unit (IMEDOS Systems UG, Jena, Germany) integrated with the RVA system. The IMEDOS software (RVA; IMEDOS Systems UG, Jena, Germany) was used to differentiate oxygenated from deoxygenated hemoglobin based on light absorption at specific wavelengths, enabling the measurement of oxygen saturation in retinal vessels. Oximetry imaging was performed at two wavelengths: 548 ± 10 nm for oxygen-insensitive imaging (green channel) and 610 ± 10 nm for oxygen-sensitive imaging (red channel) [35]. An optic disk-centered image protocol was applied, in which two concentric rings with radii of 1.0 and 1.5 optic disk diameters were drawn in the peripapillary region (Figure 1B). The area between these two rings served as the region of interest for all measurements. For each eye, four high-quality fundus images were acquired, starting with the right eye. The images were evaluated for optimal brightness using the three-channel luminance histogram tool of the RVA to minimize the effects of retinal pigmentation and brightness variability. Only images with red channel illumination <160 and green channel illumination >60 on the luminance scale were selected for further analysis. All main arterioles and venules within the defined measurement area were manually selected and analyzed [41,42].

Based on oximetry measurements, the average oxygen saturation across all four quadrants was calculated for arterioles (A-SO_2_) and venules (V-SO_2_). From these values, the arteriovenous oxygen difference (A-V SO_2_) was derived, which reflects retinal oxygen extraction. Given that oxygen saturation measurements can fluctuate in the region around the optic nerve head [40], we further assessed regional variations by comparing the overall average oxygen saturation with the values measured in the superotemporal quadrant specifically for arteries (ST A) and veins (ST V).

### 2.2. Statistical Procedure

The statistical analyses were conducted using IBM SPSS Statistics software version 21 (IBM Corp., Armonk, NY, USA). All data were confirmed to follow a normal distribution. The agreement between methods was evaluated using Bland–Altman analysis. The results were reported as adjusted means with corresponding standard deviations for controls and mean differences within subgroups, accompanied by respective *p*-values. Statistical significance was defined as *p* < 0.05.

The random factor in the analysis was defined as the “subject”, while fixed factors included “group”, “age”, “gender”, “location”, and “eye”. Study groups were treated as covariates. Independent variables analyzed included mean SO_2_ parameters (A-SO_2_, V-SO_2_, and their difference, A-V SO_2_), vessel diameter measurements (D-A and D-V), static measurements (CRA and CRV), and dynamic flicker responses (ADil and VDil). Pairwise comparisons were performed between single vessels, single images, and protocols using a paired *t*-test. Regression coefficients and corresponding *p*-values were calculated for all variables, with arterial vessel oxygen saturation values found to be comparable across different analysis approaches.

## 3. Results

The 24 participants had a mean age of 36.04 ± 14.4 years, with a gender distribution of 15 females and 9 males.

### 3.1. Retinal Oximetry

**Figure 4:**
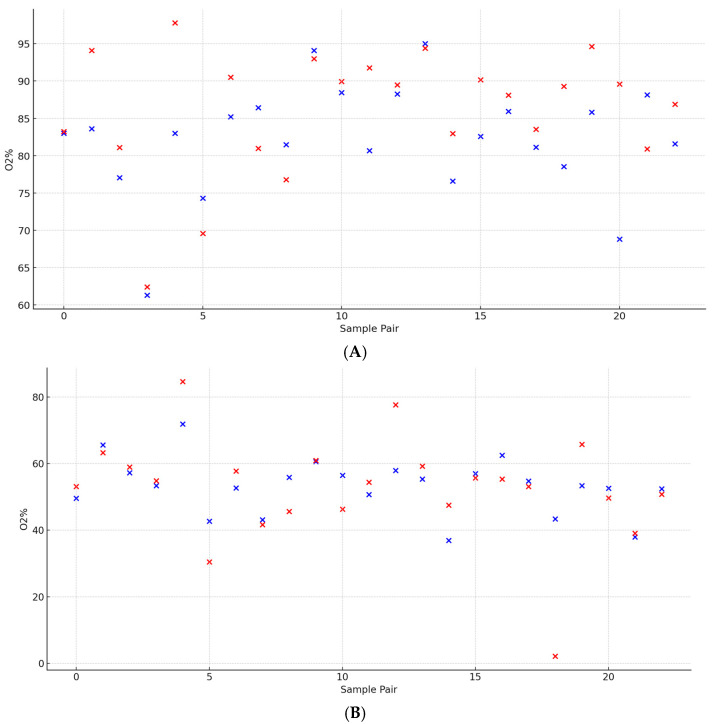
A paired analysis of retinal arteriolar vessel oxygen saturation between the superotemporal sector (ST A) and average across all sectors (A-SO_2_). (**A**) A paired analysis of retinal venous vessel oxygen saturation between the superotemporal sector (ST V) and average across all sectors (V-SO_2_) (**B**).

Mean A-SO_2_% and V-SO_2_% were 82.2 ± 7.5% and 53.2 ± 8.4% (*t* = 12.37, *p* < 0.0001), respectively, with corresponding arterial ST A and venous ST V values of 85.8 ± 8.4% and 52.2 ± 15.7% (*t* = 9.24, *p* < 0.0001). A statistically significant moderate positive correlation was observed between A-SO_2_% and ST A (*r* = 0.63, *p* = 0.0013). In contrast, V-SO_2_% showed a stronger and highly significant positive correlation with ST V (*r* = 0.70, *p* = 0.00018). The mean of the differences for A-SO_2_% and V-SO_2_% was 29.00 ± 10.00, while for ST A and ST V, it was 33.61 ± 14.98. The comparison of differences revealed a moderate positive correlation (*p* = 0.0197), suggesting a meaningful yet more variable relationship. These findings highlight both the consistency in mean relationships and the variability in differences (Figure 4).

### 3.2. Static Measurement Evaluation

**Figure 5:**
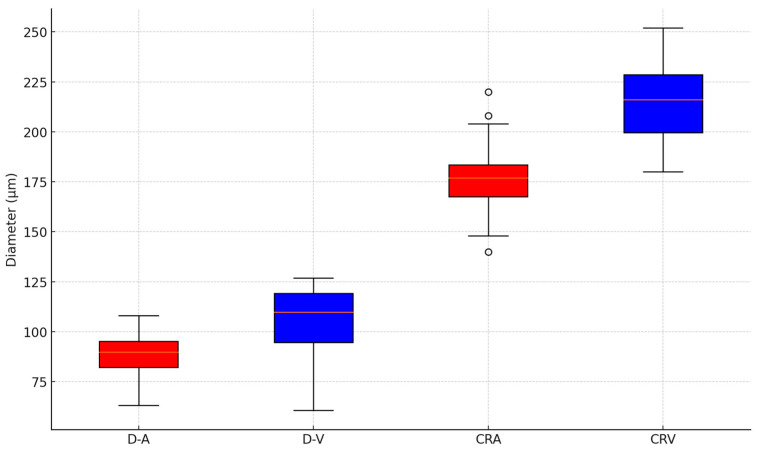
A boxplot of the central arteriolar equivalent (CRA), central venous equivalent (CRV) arteriolar diameter (D-A), and venous diameter (D-V) using static RVA analyses.

The arterial diameters (D-A) demonstrated a mean of 88.5 ± 10.8, while the venous diameters (D-V) averaged 105.2 ± 17.4. A statistical analysis revealed a significant difference between the mean arterial and venous diameters (*t* = −3.93, *p* = 0.00036). These findings illustrate a marked difference between the arterial and venous diameters, consistent with physiological expectations. The arteriovenous ratio exhibited a mean of 0.84 ± 0.06, highlighting consistency across subjects. Regarding vessel equivalents, the CRA showed a mean diameter of 178.6 ± 18.0, whereas the CRV had a larger mean diameter of 214.3 ± 18.9 (Figure 5).

### 3.3. Dynamic Flicker Light Responses

**Figure 6:**
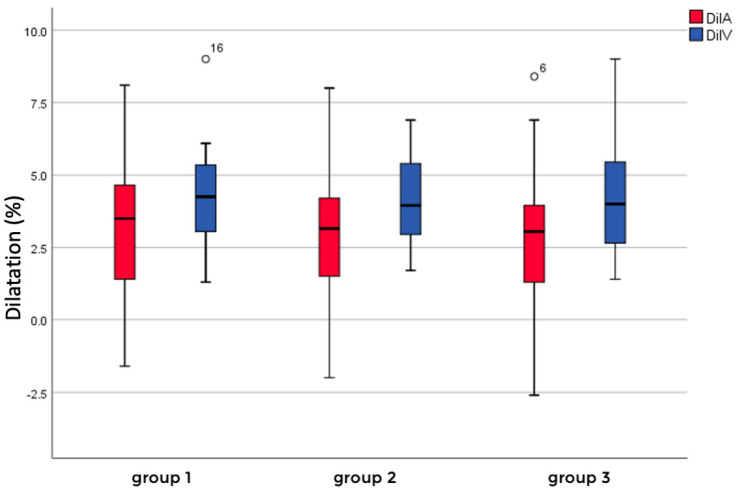
Flicker light response of arteriolar (red) and venous (blue) vessels in different groups depending on location.

The dynamic parameters reflecting the flicker light response in arterial and venous vessels were measured at three distinct retinal locations corresponding to groups I, II, and III. Arterial measurements of mean and standard deviation showed 3.36 ± 2.31 for group I, 2.97 ± 2.40 for group II, and 2.84 ± 2.29 for group III, while venous measurements consisted of 4.22 ± 1.69 for group I, 4.08 ± 1.55 for group II, and 4.21 ± 2.03 for group III. These variations highlight the structural and functional differences between arterial and venous vessels, which could influence their dynamic responses to flickering light. Additionally, a comparison between the combined groups of arterial measurements and venous measurements revealed an overall significant difference in their means (*p* = 0.0014), suggesting systematic variability between these two vessel types. However, no significant differences were observed between the individual means of the arterial measurements, with *p*-values of 0.572 for group I vs. group II, 0.437 for group I vs. group III, and 0.845 for group II vs. group III, indicating comparability across these categories. Similarly, no significant differences were identified between the individual venous measurements, with *p*-values of 0.777 for group I vs. group II, 0.988 for group I vs. group III, and 0.812 for group II vs. group III, confirming the consistency of these values. Further statistical analysis indicated that the differences between arterial and venous pairs, specifically group I (*p* = 0.150) and group II (*p* = 0.064), were not significant. However, there was a significant difference between arterial and venous responses in group III (*p* = 0.033), based on a significance level of 0.05, suggesting specific variability in this comparison. These findings provide valuable insights into the differential dynamics of arterial and venous responses to flickering stimuli in different locations (Figure 6).

### 3.4. Correlation of Dynamic, Static, and Metabolic Data

**Figure 7:**
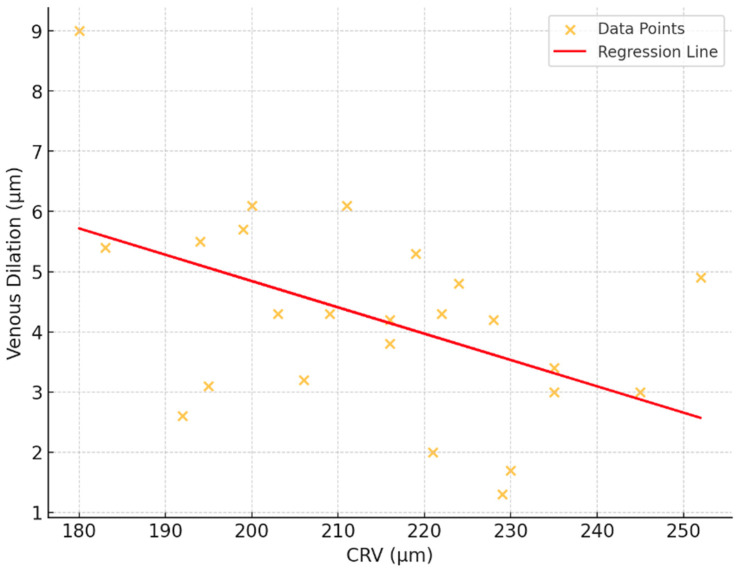
Correlation between retinal venous dilatation and central venous equivalent (CRV).

A regression analysis was conducted to explore the relationship between CRV and venous dilatation, which represent key parameters in vascular measurements. The analysis revealed a moderate negative association, with an R^2^ value of 0.239, indicating that approximately 23.9% of the variation in venous flicker responses could be explained by CRV. The regression model yielded a statistically significant result (*p* = 0.015), suggesting that changes are meaningfully associated with variations. The model’s coefficients further supported this relationship, with an intercept of 13.585 (*p* = 0.001), reflecting the estimated baseline value of venous dilatation when CRV is zero. The slope of the regression line was −0.0437 (*p* = 0.015), indicating that for each unit increase, the value decreases by approximately 0.044 units (Figure 7).

We conducted a regression analysis to investigate the relationship between the arteriolar dilation parameter, and the difference between A-SO_2_ and V-SO_2_, representing a key measure of oxygen extraction. The oxygen extraction fraction (OEF) is a crucial physiological parameter that quantifies the proportion of oxygen extracted from arterial blood as it passes through the tissue. In this study, we calculated the OEF as the ratio of the arteriovenous oxygen difference to the arterial oxygen saturation for each subject. Specifically, the OEF was determined using the following formula:

OEF = (A-SO_2_-V-SO_2_)/(A-SO_2_)

**Figure 8:**
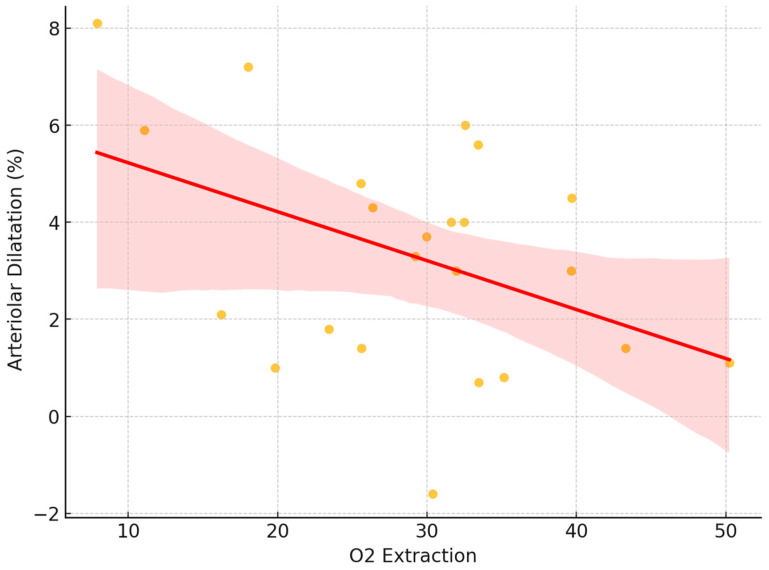
Correlation between retinal arteriolar dilatation and oxygen extraction, based on the difference in arteriolar and venous values.

The analysis revealed an average OEF of 0.3488 ± 0.11, indicating that approximately 35% of the oxygen in arterial blood was extracted by tissues on average. A linear regression analysis revealed a statistically significant association between oxygen extraction and arteriolar dilatation, with a negative slope coefficient (*p* = 0.041). The adjusted coefficient of determination (R^2^_a_dj = 0.145) indicates that approximately 14.5% of the variance in arteriolar dilatation can be explained by the oxygen extraction difference. These findings provide evidence of a significant linear relationship between oxygen extraction and arteriolar dilation (Figure 8).

## 4. Discussion

The study addresses the challenge that different methods and protocols for clinically assessing patients often vary. While this variability makes it difficult to compare studies, it is a necessary step toward developing standardized examination and analysis protocols. The primary goal of the study was to evaluate the reproducibility and sensitivity of retinal vessel diameter measurements using the Retinal Vessel Analyzer, ensuring reliable and consistent results for clinical practice. In all three groups, venules consistently exhibited a more pronounced response to flicker light stimulation compared to arterioles, confirming the differential vascular behavior [43]. This disparity was significant (*p* = 0.0014), emphasizing the potential for venular responses to serve as a sensitive marker in assessing vascular health. Our findings are consistent with existing research on the flicker light response of the retinal arterial and venous systems [26,32,44,45]. It is important to note that the vessels exhibit different patterns of behavior: following arterial dilation, constriction occurs more rapidly, whereas in veins, constriction is slower. Another significant factor is the size of the vessels, with smaller vessels within the arterial and venous networks demonstrating a greater degree of dilation [46].

While the flicker responses across the three different measurement groups showed variability, they did not reach statistical significance. This suggests that vessel location and measurement length have minimal impact on the observed dynamics under controlled conditions. This finding allows measurements to be conducted in patients who are difficult to examine without the concern that selecting a short vessel segment would compromise the validity or comparability of the results. It also contradicts the assumption that short vessel segments, in conjunction with eye movements, lead to greater measurement errors due to local variations in vessel diameter along the vessel path [44,47]. The arteriovenous differences were not statistically significant in the first and second groups. However, in the third group, which was assessed within the VesselMap region, a significant difference in flicker response between arteries and veins was observed. This finding aligns with recommendations from other studies [40,48] suggesting that measurements within the VesselMap region, approximately 0.5–1 OD, may offer higher precision. This improved accuracy could result from minimizing artifacts such as vessel crossings or closely adjacent arteries and veins, which might otherwise obscure the detection of such differences in other regions.

Retinal oximetry provides a unique approach to assessing retinal vascular health by quantifying oxygen saturation levels in arterioles and venules. In our study, the mean oxygen saturation levels were 82.2% in arterioles and 53.2% in venules. These measurements, particularly the arterial values, were slightly lower than those reported in the literature [49] but exhibited significant interindividual variability. The study population consisted of relatively young participants (mean age 36.04 ± 14.4 years) and healthy individuals without relevant cardiovascular or other systemic diseases. However, data regarding participants’ smoking status or cardiorespiratory fitness were not collected, representing potential confounding factors. Another minor factor influencing oxygen saturation levels is the altitude of our facility, located at 650 m above sea level. While this altitude has a relatively small impact on oxygen saturation, increasing altitude is known to induce physiological adaptations over time [50]. Our analysis found no significant difference between the oximetry values measured in the superotemporal quadrant and the average values across all quadrants. This supports the validity and reliability of these measurements across the examined retinal regions.

Our findings highlight the significance of flicker-induced vascular responses as potential biomarkers for systemic diseases. While no direct causal relationship was observed between the metabolic parameters of arteries or veins and their respective flicker light responses, we identified a significant negative correlation between arterial dilation during flicker stimulation and both the oxygen extraction fraction (OEF) and the arteriovenous oxygen difference (AV-SO_2_).

The OEF in our patients was 0.3488 ± 0.11, indicating that approximately 35% of the oxygen available from the retinal circulation was extracted for energy metabolism by the inner retinal tissue. This value is consistent with findings from other studies [51], such as an OEF of 0.44 in the human brain [52] and 0.46 in the inner retina of rats [53] observed in experimental models. The arteriovenous oxygen difference (AV-SO_2_), a measure of the retinal metabolic oxygen consumption, further underscores the metabolic demand of the inner retina. Our data suggest that high oxygen extraction, resembling a hypoxia-like state, is associated with increased arterial dilation. This physiological behavior is well documented in cerebral vasculature and is believed to facilitate an increase in blood flow to meet the metabolic demands [54,55].

Additionally, the observed increase in retinal arteriovenous oxygen extraction during flicker light stimulation supports the hypothesis that the inner retinal metabolic demand is a primary driver of the autoregulatory vascular response. This response aligns with previous studies suggesting that elevated metabolic activity triggers an increase in oxygen supply and utilization. Such mechanisms are likely mediated by neurovascular coupling and nitric oxide (NO)-dependent pathways [56,57,58]. Collectively, these findings contribute to a deeper understanding of the interplay between retinal oxygen metabolism and vascular regulation, offering insights into both normal physiology and pathological states.

Venous vessels are capable of dilating in response to flicker light stimulation; however, the relationship between their baseline diameter and dilation capacity is not as well defined as it is for arteries [59]. This difference can be attributed, in part, to the structural composition of veins, which have a less prominent muscular layer in the tunica media compared to arteries [60]. In our study, we identified a significant correlation between venous dilation and central retinal vein (CRV) diameter. Specifically, we observed that venous dilation induced by flicker light decreases as CRV diameter increases. According to the Hagen–Poiseuille law, blood flow is proportional to the fourth power of the vessel diameter; therefore, such an increase in CRV diameter suggests an augmentation of retinal blood flow.

When the capacity for vascular dilation is limited, intravascular pressure rises, triggering autoregulatory mechanisms to maintain blood flow. This autoregulation is primarily mediated by the Bayliss effect, a myogenic, stretch-induced contractile response to increased intraluminal pressure that counteracts further dilation [61,62]. While these physiological mechanisms provide a partial explanation for the observed correlations, they have predominantly been studied in arterial systems. Further investigation is required to fully elucidate the interplay of these dynamics within venous systems.

The ability of flicker light stimulation to non-invasively assess vascular dynamics offers a unique window into retinal and microvascular health. This makes it a valuable tool for understanding microvascular regulation and detecting early pathophysiological changes associated with various medical conditions. The observed relationships between static vessel properties, dynamic responses, and metabolic parameters underscore the complexity of these interactions and their potential diagnostic value.

Future research should focus on refining experimental methodologies to reduce variability and improve reproducibility, as well as exploring the underlying mechanisms in both arterial and venous systems. This would enhance our understanding of disease progression and provide a framework for evaluating the efficacy of therapeutic interventions aimed at microvascular dysfunction.

## Data Availability

The original contributions presented in this study are included in the article. Further inquiries can be directed to the corresponding author.

## Abbreviations

The following abbreviations are used in this manuscript:

ADil	Arterial dilation
A-SO_2_	Oxygen saturation of arterioles
A-V-SO_2_	Arterio-venular difference
AVR	Arteriovenous ratio
CRA	Central retinal arteriolar equivalent
CRV	Central retinal venous equivalent
D-A	Retinal vessel diameter of arteries
D-V	Retinal vessel diameter of veins
DVA	Dynamic retinal vessel analysis
FL	Flicker light
IOP	Intraocular pressure
MAD	Mean arterial diameter
MVD	Mean venous diameter
OD	Optic disk
OEF	Oxygen extraction fraction
RFR	Retinal vessel flicker response
RO	Retinal vessel oximetry
RPE	Retinal pigment epithelium
RVA	Retinal Vessel Analyzer
ST	Superotemporal area
ST A	Arteriolar superotemporal area
ST V	Venous superotemporal area
StO2	retinal tissue oxygenation
SVA	Static retinal vessel analysis
VDil	Venous dilation
V-SO_2_	Oxygen saturation of venules
